# Flexural properties of rapidly prototyped denture base materials: the effect of nanoparticle addition and post-curing duration *in vitro*

**DOI:** 10.3389/fdmed.2025.1544474

**Published:** 2025-02-24

**Authors:** Shaimaa M. Fouda, Mohammed M. Gad, Mai El Zayat, Soban Q. Khan, Sultan Akhtar, Ahmed Othman, Constantin von See

**Affiliations:** ^1^ Department of Substitutive Dental Sciences, College of Dentistry, Imam Abdulrahman Bin Faisal University, P.O. Box 1982, Dammam, Saudi Arabia; ^2^ Department of Dental Education, College of Dentistry, Imam Abdulrahman Bin Faisal University, P.O. Box 1982, Dammam, Dammam, Saudi Arabia; ^3^ Department of Biophysics, Institute for Research and Medical Consultations (IRMC), Imam Abdulrahman Bin Faisal University, P.O. Box 1982, Dammam, Saudi Arabia; ^4^ Research Center for Digital Technologies in Dentistry and CAD/CAM, Department of Dentistry, Faculty of Medicine and Dentistry, Danube Private University, Krems, Austria

**Keywords:** CAD-CAM, nanoparticles, post-curing duration, mechanical testing, complete denture

## Abstract

**Objectives:**

The flexural strength and elastic modulus of rapidly prototyped denture base materials are affected by numerous factors including reinforcement with nanoparticles (NPs) and post-curing duration (PCD), though the effect of these two factors together has been overlooked. The present study tested the effect of nanodiamonds (NDs) or silicon dioxide nanoparticles (SNPs) with various PCDs on the flexural strength and elastic modulus of rapidly prototyped denture base materials.

**Methods:**

To measure the flexural strength and elastic modulus, bar-shaped specimens (64 × 10 × 3.3 mm) were designed and rapidly prototyped using ASIGA and NextDent denture base resins. Each resin (*N* = 150) was divided into five groups (*n* = 30) according to NP type and concentrations: pure group as a control without additives, 0.25% NDs, 0.5% NDs, 0.25% SNPs, and 0.5% SNPs. Specimens from each group were further divided into three groups (*n* = 10) and post-cured for 15, 60, or 90 min, followed by thermocycling for 5,000 cycles. After measuring the flexural strength and elastic modulus using a three-point bending test, a scanning electron microscope was used to analyze the fractured surface. The bonds between the NPs and the resin were tested by Fourier-transform infrared spectroscopy. ANOVA and *post hoc* tests were used for data analysis (*α* = 0.05).

**Results:**

The flexural strength increased with prolonged PCD and the highest values for all tested groups were reported at 90 min (*P* < 0.001). The flexural strength of both materials increased significantly with the addition of NDs and SNPs in comparison to the pure groups (*P* < 0.05). *K*-factor ANOVA analysis of the elastic modulus showed that each factor (NP type, PCD, and material type) had a significant effect on the elastic modulus (*P* < 0.001).

**Conclusion:**

The flexural strength and elastic modulus of rapidly prototyped denture base resin were increased with the addition of NDs or SNPs and when increasing the PCD. Factors including nanoparticle type and concentration, the post-curing duration, and the material type solely or in combination could affect the flexural strength and elastic modulus of prototyped denture base materials.

## 1 Introduction

Dentures are subjected to multiple intraoral forces that could cause denture fracture or deformation if the denture base material has low flexural properties. Therefore, the materials used for denture fabrication must possess acceptable strength to ensure long-term clinical performance (1, 2). Polymethyl methacrylate (PMMA) is the most common material used for denture base fabrication due to its advantages such as esthetics, affordable price, and ease of fabrication and repair (3). However, some disadvantages have been reported including low flexural strength (FS), elastic modulus (EM), and impact strength which are considered as the main causes of denture fracture (4, 5). Due to the continuous stress that the denture base is subjected to, the occurrence of denture fracture is approximately 64%–68% within 3 years of clinical use (5, 6). Therefore, attempts have been made to overcome these drawbacks by using new materials and technologies for denture fabrication, in addition to reinforcement of denture base materials with different additives (7, 8).

Denture fabrication using computer-aided designing and manufacturing (CAD-CAM) technologies is more prevalent due to multiple advantages such as reduced fabrication time and increased denture accuracy and patient satisfaction (9). Using the additive fabricating method, also called rapid prototyping or 3D printing, for denture fabrication is less expensive than using the subtractive (milling) method in addition to having higher accuracy and using less material (10). However, the milled resin has superior mechanical properties compared to the 3D-printed resin (11, 12). There are different factors that could increase the strength of rapidly prototyped (RP) resin, amongst these is nanofiller addition (7, 13). The printing technology, material composition and post-curing duration (PCD) can also affect the resin's mechanical behavior (14, 15). The addition of nanoparticles (NPs) to PMMA and rapidly prototyped resin has been investigated in previous studies in an attempt to enhance the resin's mechanical performance and decrease microbial colonization (8, 13). It was reported that the addition of nanoparticles to PMMA improved the strength and surface properties and the antimicrobial efficacy compared with unmodified resin. Thus PMMA/nanocomposites are recommended for denture base fabrications (3, 16, 17). Recent reviews stated that the addition of nanoparticles to 3D-printed resins resulted in high performance compared with unmodified resins (13, 18). This improvement in the properties of the resin with the introduced nanocomposites highlights the importance of nanotechnology application in combination with the new CAD-CAM technologies for denture fabrications (13, 18).

The inclusion of nanodiamonds (NDs) or silicon dioxide nanoparticles (SNPs) to heat-polymerized PMMA improved the resin’s mechanical performance (19, 20). A similar effect was found with the addition of SNPs to prototyped resin, as reported by Gad et al. (7). Mangal et al. (21) found the addition of 0.1 wt% NDs increased the strength of rapidly prototyped orthodontic appliances. Moreover, NDs have shown an antimicrobial effect and a reduction of *Candida albicans* adhesion (22, 23). The thermal conductivity of denture resin was also increased with the addition of a small amount of NDs (24).

The predominant technologies employed in the additive fabrication of dental prostheses are stereolithography and the digital light processing method (25, 26). After printing, the prototyped objects are subjected to a post-curing process to ensure complete polymerization of the resin (13). The post-curing process and variables such as the curing unit, light intensity, and PCD affect the resin's characteristics (13). Previous studies have investigated the influence of PCD on the flexural strength of prototyped resin and reported increased flexural strength with prolonged curing time, while other studies found no correlation (15, 27–30).

However, the influence of the addition of NDs or SNPs and PCD on the flexural strength and elastic modulus of RP denture base resins has not been tested before. Thus, the purpose of this study was to assess the effects of adding NDs or SNPs to RP denture base resins on their elastic modulus and flexural strength with different PCDs. The study’s null hypotheses stated that the flexural strength or elastic modulus of RP resin would not change with (a) nanoparticle addition, (b) post-curing duration, or (c) the combined effect of nanoparticle addition and post-curing duration.

## 2 Materials and methods

The number of required specimens was calculated according to the findings of a previous study, indicating the need for 300 specimens (150/resin, 50/NP, 30/concentration, 10/posturing time) (31). Each RP denture base resin (ASIGA and NextDent) (*N* = 150) was divided into five groups (*n* = 30) according to NP type and concentrations: a control group of pure resin without additives, 0.25% NDs, 0.5% NDs, 0.25% SNPs, and 0.5% SNPs. Each concentration group was further divided (*n* = 10) according to PCD (15, 60, and 90 min).

The tested RP denture base resins were NextDent (Denture 3D + NextDent B.V., Soesterberg, The Netherlands) printed using a NextDent 5100, and ASIGA (DentaBASE, ASIGA, Erfurt, Germany) printed using a ASIGA MAX™ and digital light processing technology.

For nanocomposite preparation, the NDs (Shanghai Richem International Co. Ltd, Shanghai, China) were heat treated at 450°C for 2 h in air as described in previous studies (32, 33). The SNPs (AEROSIL R812; Evonik Degussa, Germany) were silanated using a silane coupling agent [3-trimethoxysilyl propyl methacryate, 97% (γ-MPS)] using the same method detailed in Gad et al.’s study (7). For nanomixture preparation, each resin was shaken on a shaker according to the manufacturer’s recommendations followed by NP weighting and the addition of 0.25% or 0.5% wt concentrations. Each container was shaken again to ensure the NPs were well-distributed in the resin matrix.

The ISO standards (ISO 20795-1:2013) (34) for flexural strength and elastic modulus testing were followed. The specimens were designed in 64 × 10 × 3.3 mm dimensions using AutoCAD software (123D design, Autodesk, version 2.2.14, San Rafael, USA) and then transformed into standard tessellation language files that were imported to the corresponding printer. The printing orientation was set at a 90° angle and a layer thickness of 50 µm. The prototyped specimens were washed with isopropyl alcohol (99.9%) and then post-cured in the corresponding post-curing unit, NextDent specimens in an LC-D Print Box and ASIGA specimens in an ASIGA-Flash, for 15, 60, or 90 min. After support removal, the specimens were polished in moist conditions for 5 min at 100 rpm using 1,200-grit sandpaper (MicroCut PSA; Buehler, IL, USA) in a polishing machine (Metaserv 250 grinder-polisher; Buehler GmbH, IL, USA). The specimens were then kept in distilled water at 37°C for 2 days and subjected to 5,000 thermal cycles (5 and 55°C/30 s) using a thermocycling machine (THE-1100 Thermocycler, SD Mechatronik Thermocycler, Germany).

A universal testing machine (Instron Model 8871; Instron Corp., Norwood, MA, USA) was employed to evaluate the flexural strength and elastic modulus using a three-point bending test according to the ISO standard (ISO 20795-1:2013) (34). The specimens were subjected to a 5 kN load with an across-head speed of 5 mm/min at the center between two vertical supports at a 50 mm distance. The fracture load (N) was noted to calculate the flexural strength (MPa) and elastic modulus (GPa) according to the equations FS = 3 WL/2*bh*^2^ and EM = FL^3^/4*bh*^3^*d*, respectively, as described in previous studies (7, 31).

A scanning electron microscope (SEM; INSPECT S50, FEI, Czech Republic; 20 kV) was employed to study the fractured surfaces. The specimens were mounted onto metallic stubs and were gold coated using a sputter coating machine. The micrographs were taken under different magnifications in order to determine the fracture type and to highlight the distribution of nanoparticles.

**Figure 1:**
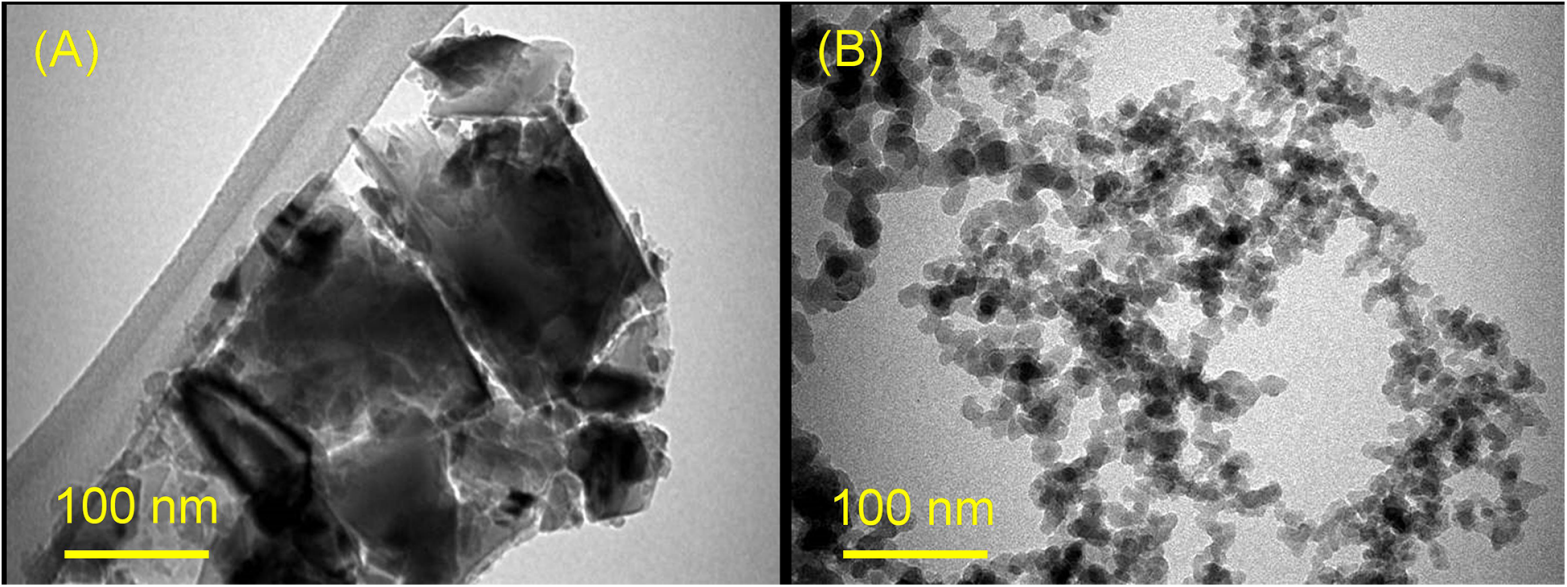
TEM images of NDs **(A)** and SNPs **(B).** The scale bars are 100 nm.

Transmission electron microscopy (TEM; Morgagni 268, FEI; operated at 80 kV) was used to detect the nanoparticles’ (NDs and SNPs) size and morphology. The sample suspensions were placed onto TEM copper grids with carbon films and several TEM images were acquired (Figure 1).

The bonds of the NDs and SNPs within the resin were inspected using Fourier-transform infrared (FTIR) spectroscopy using a transmission spectroscope (Hartmann & Braun, MB-series). The FTIR spectra were measured by scanning the specimens between the 4,000 and 400 cm^–1^ wavenumber regions.

A descriptive analysis of the data was presented by calculating the mean and standard deviation of the tested properties. The normal distribution of the data was tested with the Shapiro–Wilk test and the non-significant results showed that the data were normally distributed. Inferential data analysis was conducted using parametric tests. The effect of one factor (concentration, time, etc.) on the tested properties was tested by one-way ANOVA. Tukey’s *post hoc* test was used for the pairwise comparison. Due to having more than three factors in the study and to evaluate the interacting effects of all these factors, *k*-factor ANOVA was used. Two-independent samples *T*-test was employed to study the influence of NP concentration (0.25% and 0.5%) on the tested properties. *P*-values less than 0.05 were statistically significant.

## 3 Results

Table 1 shows the effect of PCD per concentration on the flexural strength of the test groups. The variation caused by the PCD on the flexural strength in each NP concentration level for the NextDent and ASIGA resins was statistically significant (*P* < 0.001). For all groups, the flexural strength increased as the PCD increased and a 90-min PCD resulted in the significantly (*P* < 0.001) highest FS when compared with 15- and 60-min groups per respective NP type and concentrations. For the 0% concentration, all the pairwise comparisons of the NextDent and ASIGA resins were found to be statistically significant. For 0.25% and 0.5% concentrations of NDs, pairwise comparisons for each material showed a statistically significant difference in means. However, in the case of NextDent resin with 0.25% and 0.5% SNP concentrations, a pair (15 min vs. 60 min) showed no significant difference in means (*P* = 0.539 and 0.998, respectively).

**Table 1:** Effect of post-curing duration and nanoparticle concentration on the flexural strength of the tested materials.

Tested property	Material	NP	Conc.	Post-curing duration			
				15 min	60 min	90 min	*P*-value
Flexural strength (MPa)	NextDent	PURE	0%	72.3 (2.8)	79.9 (2.6)	85.2 (2.6)	<0.001*
		NDs	0.25%	78.9 (7.0)^A^	89.1 (2.6)^A^	99.3 (0.7)	<0.001*
			0.5%	81.1 (2.8)^A^	92.7 (5.2)^A^	102.6 (3.3)	<0.001*
		*P*-value		0.001*	<0.001*	<0.001*	
		PURE	0%	72.3 (2.8)	79.9 (2.6)	85.2 (2.6)	<0.001*
		SNPs	0.25%	112.8 (4.1)^a^	115.7 (7.6)^a^	126.3 (6.1)^A^	<0.001*
			0.5%	107.7 (5.2)^a^	107.9 (7.4)^a^	121.9 (5.4)^A^	<0.001*
		*P*-value		<0.001*	<0.001*	<0.001*	
	ASIGA	PURE	0%	73.3 (2.7)^A^	81.1 (2.4)^A^	87.3 (3.2)	<0.001*
		NDs	0.25%	74.7 (3.0)^A^	83.1 (4.7)^A,B^	90.7 (2.4)^A^	<0.001*
			0.5%	79.2 (2.8)	85.5 (1.8)^B^	92.7 (2.6)^A^	<0.001*
		*P*-value		<0.001*	0.019*	0.001*	
		PURE	0%	73.3 (2.7)	81.1 (2.4)	87.3 (3.2)	<0.001*
		SNPs	0.25%	90.2 (3.7)	109.4 (6.4)	119.8 (4.1)	<0.001*
			0.5%	83.9 (2.3)	92.7 (3.4)	101.2 (3.8)	<0.001*
		*P*-value		<0.001*	<0.001*	<0.001*	

The same lowercase letter in each row for each material denotes an insignificant difference between the pairs.

The same uppercase letter in each column for each material denotes an insignificant difference between the pairs.

* Statistically significant at a 0.05 level of significance.

The influence of nanoparticle concentration on flexural strength at each PCD was also analyzed (Table 1). In the case of NDs, both concentrations significantly increased the flexural strength compared with the unmodified groups (*P* = 0.000 and 0.000) except for the ASIGA resin when modified with 0.25% NDs at 15 and 60 min (*P* = 0.539 and *P* = 0.35). In the ND groups, NextDent resin with 0.5% NDs and 90-min PCD had the significantly highest flexural strength value (102.6 ± 3.3 MPa). For ASIG resin, no significant difference was seen between ND concentrations at 60 and 90 min (*P* = 0.254 and *P* = 0.234). However, ASIGA resin with 0.5% NDs and 90-min PCD had the highest flexural strength value (92.7 ± 2.6 MPa).

In the case of SNP addition to both resins, both concentrations significantly increased the flexural strength compared with the unmodified groups (*P* < 0.001). Regardless of the resin type, 0.25% SNPs had the highest flexural strength values when compared with the 0.5% group (*P* < 0.001), except for NextDent resin with 0.25% SNPs and 90 min PCD vs. 0.5% SNPs, which showed no significant difference (*P* = 0.129).

*K*-factor ANOVA analysis for flexural strength showed that each factor (NP type and concentration, PCD, and material type) significantly affected flexural strength (*P* < 0.001). In addition, all the interacting effects of two factors were statistically significant and the interaction effects of three variables were also found to be significant. However, the interaction of all four factors was not significant (Table 2).

**Table 2:** K-factor ANOVA for multiple factors' effects on flexural strength.

	Sum of squares	DF	Mean square	*F*-value	*P*-value
Intercept	3,020,742.666	1	3,020,742.666	191,979.769	<0.001*
Time	16,601.521	2	8,300.761	527.545	<0.001*
Concentration	463.738	1	463.738	29.472	<0.001*
NP type	48,792.272	2	24,396.136	1,550.468	<0.001*
Material type	4,265.258	1	4,265.258	271.073	<0.001*
Time×concentration	102.542	2	51.271	3.258	0.040*
Time×NP type	410.061	4	102.515	6.515	<0.001*
Time×material type	142.285	2	71.143	4.521	0.012*
Concentration×NP type	2,680.965	2	1,340.482	85.193	<0.001*
Concentration×material type	165.527	1	165.527	10.520	0.001*
NP type×material type	4,508.649	2	2,254.324	143.271	<0.001*
Time×concentration×NP type	163.613	4	40.903	2.600	0.036*
Time×NP type×material type	908.477	4	227.119	14.434	<0.001*
Concentration×NP type×material type	331.217	2	165.608	10.525	<0.001*
Time×concentration×material type	121.169	2	60.584	3.850	0.022*
Time×concentration×NP type×material type	118.833	4	29.708	1.888	0.112
Error	5,098.040	324	15.735		
Total	3,105,616.832	360			

* Statistically significant at the 0.05 level of significance.

The elastic modulus mean values and standard deviations of the tested resins regarding the effect of PCD and nanoparticle concentration are summarized in Table 2. The elastic modulus was significantly increased with an increase in PCD for NextDent resin with 0.25% NDs (*P* < 0.001) and 0.25% SNPs (*P* = 0.007) and the highest value was found with 90-min PCD without a significant difference between 60 and 90 min. In the case of ASIGA resin, the increase in elastic modulus was significant for 0.25% NDs (*P* < 0.001) with the highest value with a PCD of 90 min, without a significant difference between 90 and 60 min. Furthermore, the ASIGA 0.5% SNP group had the highest elastic modulus at 60 min (*P* < 0.001) without a significant difference between 15 and 90 min.

The effect of nanoparticle concentration at each PCD was also analyzed (Table 3). In the case of NextDent resin with NDs, the elastic modulus was significantly decreased compared to the unmodified groups at 15 min for the 0.25% group (*P* = 0.013), at 60 min for the 0.5% group (*P* = 0.017), and at 90 min for the 0.5% group (*P* = 0.003). In the case of ASIGA resin with NDs, the elastic modulus was significantly decreased with both concentrations compared to the unmodified groups (*P* < 0.001) at each PCD. The elastic modulus of NextDent resin with SNPs at 15 min with a concentration of 0.5% was significantly increased compared to the unmodified groups (*P* < 0.001) while at 60 and 90 min, none of the concentration levels had any significant effect on the elastic modulus compared to the unmodified groups. In the case of ASIGA resin with 0.5% SNPs at 15 and 90 min, the elastic modulus was significantly decreased compared with the unmodified resin (*P* < 0.001) while at 60 min, the changes in elastic modulus with 0.25% and 0.5% were not significantly different compared with the unmodified groups (*P* = 0.926 and 0.685, respectively).

**Table 3:** Effect of post-curing duration and nanoparticle concentration on the elastic modulus of tested materials.

Tested property	Material	NP	Conc.	Post-curing duration			
				15 min	60 min	90 min	*P*-value
Elastic modulus (GPa)	NextDent	PURE	0%	3,579.9 (399.6)^A^	3,726.1 (436.2)^A^	3,928.8 (509.7)^A^	0.239
		NDs	0.25%	3,239.5 (89.4)^a,b,A^	3,563.9 (202.1)^a^	3,603.2 (153.5)^b^	<0.001*
			0.5%	3,332.9 (115.1)	3,338.2 (88.6)^A^	3,392.2 (118.5)^A^	0.411
		*P*-value		0.013*	0.017*	0.003*	
		PURE	0%	3,579.9 (399.6)^A^	3,726.1 (436.2)	3,928.8 (509.7)	0.239
		SNPs	0.25%	3,539.9 (307.7)^a,b,B^	3,875.4 (242.9)^a^	3,978.1 (355.7)^b^	0.007*
			0.5%	3,951.7 (255.8)^A,B^	3,984.7 (400.0)	3,929.4 (334.2)	0.934
		*P*-value		0.016*	0.307	0.952	
	ASIGA	PURE	0%	3,511.0 (132.5)^A,B^	3,641.3 (220.9)^A,B^	3,585.7 (156.6)^A,B^	0.262
		NDs	0.25%	2,553.8 (139.7)^a,b,A^	2,883.5 (135.9)^a,A^	2,963.7 (138.3)^b,A^	<0.001*
			0.5%	2,923.4 (264.4)^B^	2,995.2 (315.7)^B^	3,060.4 (321.7)^B^	0.603
		*P*-value		<0.001*	<0.001*	<0.001*	
		PURE	0%	3,511.0 (132.5)^A^	3,641.3 (220.9)	3,585.7 (156.6)^A^	0.262
		SNPs	0.25%	3,412.8 (332.5)^B^	3,590.8 (290.8)	3,761.1 (287.9)^B^	0.503
			0.5%	2,883.5 (135.9)^a,A,B^	3,528.3 (376.5)^a,b^	3,050.4 (171.1)^b,A,B^	<0.001*
		*P*-value		<0.001*	0.708	<0.001*	

The same lowercase letter in each row for each material denotes a significant difference between the pairs.

The same uppercase letter in each column for each material denotes a significant difference between the pairs.

* Statistically significant at the 0.05 level of significance.

*K*-factor ANOVA analysis for elastic modulus showed that each factor (NP type, PCD, and material type) had a significant effect on elastic modulus (*P* < 0.001) but the effect of concentration was not significant (*P* = 0.270). All the interacting effects of two factors except time and NP type, time and material type, and concentration and material type were also statistically significant (*P* < 0.05), while interaction effects of three variables except time, NP type, and material type; and time, concentration, and material type were found to be statistically significant (*P* < 0.05). However, the interaction of all four factors did not produce any insignificant effect on the elastic modulus (Table 4).

**Table 4:** K-factor ANOVA for multiple factors’ effects on elastic modulus.

	Sum of squares	DF	Mean square	*F*-value	*P*-value
Intercept	4,359,864,896.038	1	4,359,864,896.038	53,927.543	<0.001*
Time	3,820,948.982	2	1,910,474.491	23.631	<0.001*
Concentration	98,568.824	1	98,568.824	1.219	0.270
NP type	19,204,536.785	2	9,602,268.392	118.771	<0.001*
Material type	14,065,695.128	1	14,065,695.128	173.980	<0.001*
Time×concentration	640,874.059	2	320,437.029	3.964	0.020*
Time×NP type	342,706.642	4	85,676.660	1.060	0.376
Time×material type	283,629.385	2	141,814.693	1.754	0.175
Concentration×NP type	521,772.556	2	260,886.278	3.227	0.041*
Concentration×material type	202,360.583	1	202,360.583	2.503	0.115
NP type×material type	2,376,122.518	2	1,188,061.259	14.695	<0.001*
Time×concentration×NP type	821,704.862	4	205,426.216	2.541	0.040*
Time×NPs×material type	494,571.412	4	123,642.853	1.529	0.193
Concentration× NP type×material type	3,130,071.150	2	1,565,035.575	19.358	<0.001*
Time×concentration×material type	293,578.062	2	146,789.031	1.816	0.164
Time×concentration×NP type×material type	469,626.832	4	117,406.708	1.452	0.217
Error	26,194,336.957	324	80,846.719		
Total	4,432,826,000.773	360			

* Statistically significant at the 0.05 level of significance.

**Figure 2:**
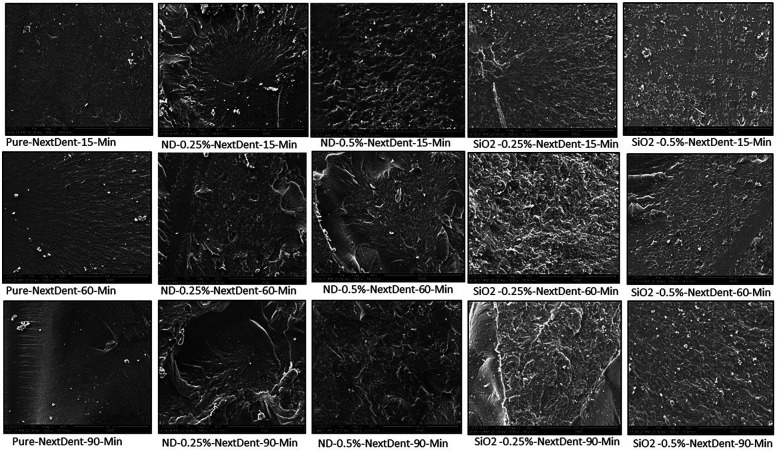
Representative SEM images with 1,000× magnification of fractured NextDent specimens with added nanoparticles at different PCDs.

**Figure 3:**
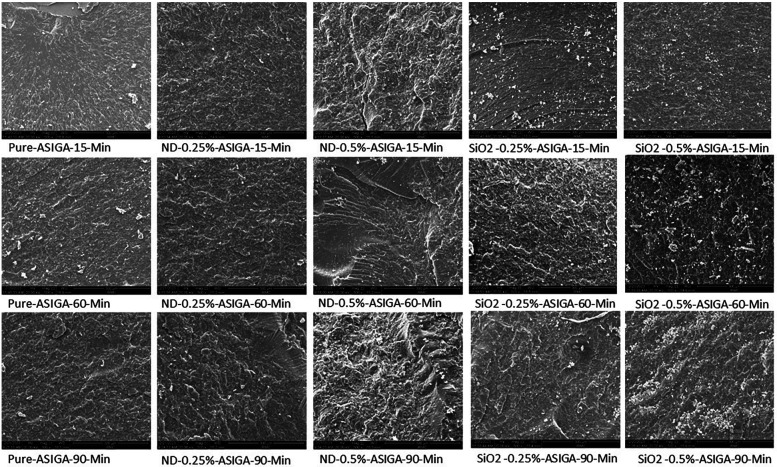
Representative SEM images with 1,000× magnification of fractured ASIGA specimens with added nanoparticles at different PCDs.

Figures 2, 3 show the SEM analysis of the fractured surfaces of NextDent and ASIGA resins, respectively. For the pure resins, the fractured surface had a smooth surface with an absence of irregularities in NextDent resin (Figure 2) and a slightly faint irregularity in ASIGA resin (Figure 3). Pure NextDent resin had the same features with increased PCD while in pure ASIGA, the lamellae appeared and irregularity increased as PCT increased. With NP addition, the surface topography and features changed from smooth to irregular with infirm lamellae, representing the ductile fracture type. Regarding NP type, both concentrations had the same features with sharp and deep lamellae which slightly increased with ND concentration. For SNPs, there were faint lamellae with some clustering in the 0.5% concentration group compared with 0.25%.

**Figure 4:**
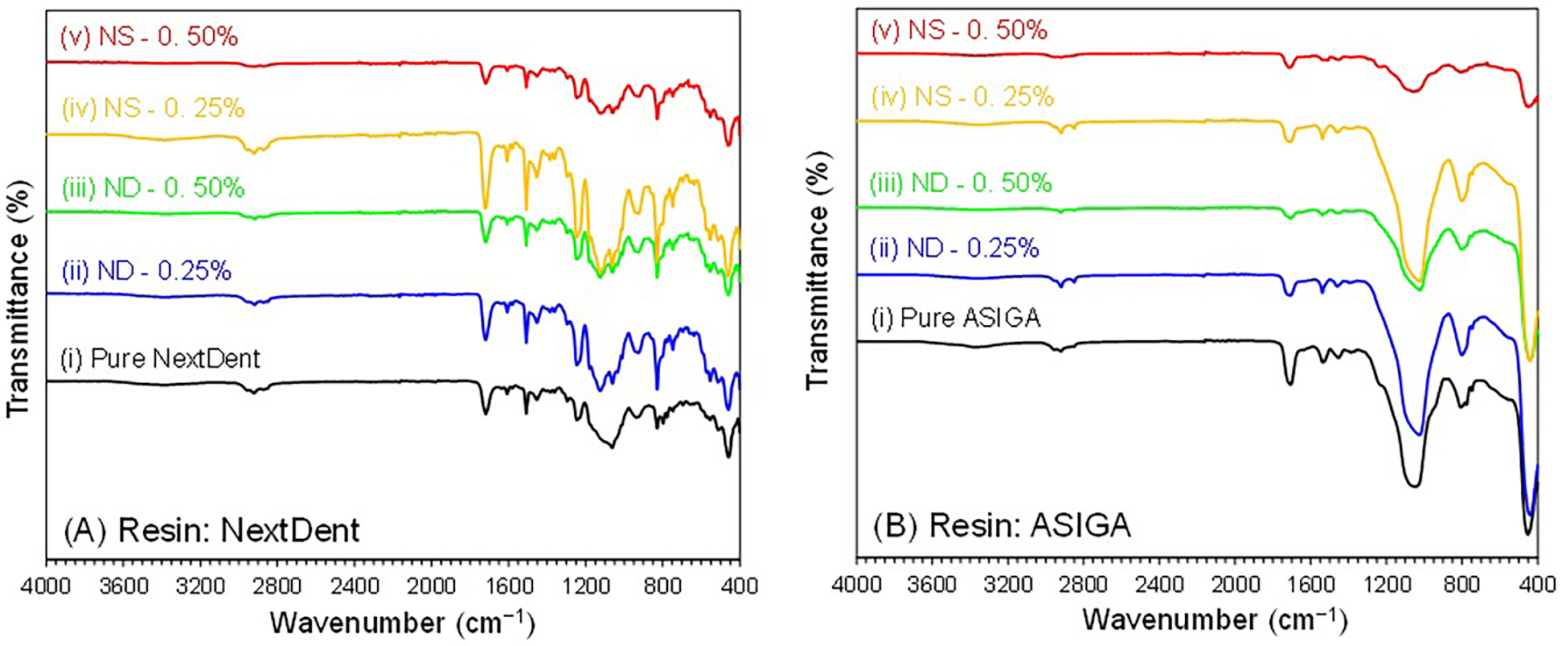
FTIR spectra of pure and NPs incorporated **(A)** NextDent and **(B)** ASIGA specimens (60 min). NextDent specimens: pure NextDent-60-min, NDs-0.25%-NextDent-60-min, NDs-0.50%-NextDent-60-min, SNPs-0.25%-NextDent-60-min, and SNPs-0.50%-NextDent-60-min. ASIGA specimens: pure ASIGA-60-min, NDs-0.25%-ASIGA-60-min, NDs-0.50%-ASIGA-60-min, SNPs-0.25%-ASIGA-60-min, and SNPs-0.50%-ASIGA-60-min.

The FTIR spectra of the pure and modified prototyped resins (NextDent and ASIGA) with NDs and SNPs showed similar types of bands and characteristic features. For simplicity, specimens that were post-cured for 60 min were chosen for FTIR analysis for both resins (NextDent and ASIGA) (Figure 4). The studied NextDent resins specimens were referred to as (i) pure NextDent-60 min, (ii) NDs-0.25%-NextDent-60-min, (iii) NDs-0.50%-NextDent-60-min, (iv) SNPs-0.25%-NextDent-60-min, and (v) SNPs-0.50%-NextDent-60-min. Similarly, ASIGA specimens were named (i) pure ASIGA-60-min, (ii) NDs-0.25%-ASIGA-60-min, (iii) NDs-0.50%-ASIGA-60-min, (iv) SNPs-0.25%-ASIGA-60-min, and (v) SNPs-0.50%-ASIGA-60-min. The FTIR results prove that the incorporation of NPs did not alter the chain structures of the nanocomposites and only varied the intensity of the bands. The characteristic bands for each specimen are shown in Figure 4.

## 4 Discussion

The influence of the addition of NDs or SNPs with different PCDs on the flexural strength and elastic modulus of prototyped denture base resins was tested in this study. The study’s null hypotheses were rejected because nanoparticle addition, increasing the PCD, and the combined effect of both variables affected the flexural strength and elastic modulus of the tested rapidly prototyped resins.

The flexural strength of both tested materials increased with the addition of NDs or SNPs. Previous studies that have tested the impact of NDs on the flexural strength of PMMA and rapidly prototyped resin showed similar findings (19, 21, 33). Mangel et al. (21) found an increase in the flexural strength and elastic modulus of 3D-printed orthodontic appliances with the addition of NDs at 0.1% wt. Al Harbi et al. (33) tested the effect of ND addition to conventional PMMA at concentrations ranging from 0.5 to 0 1.5% wt and found the highest flexural strength at 0.5% wt. The reason for the increased flexural strength of prototyped resin with ND addition could be the high strength and surface characteristics of NDs (35). Another factor that could lead to the higher strength of the nanocomposite is adequate bonding between NDs and the resin matrix. NDs undergo heat treatment for purification which results in the creation of reactive surface carboxyl and hydroxyl groups that improve bonding with the resin matrix (32, 36). The results showed that the flexural strength increased at higher ND concentrations (0.5% than 0.25%) but the difference between them was significant only with NextDent at 90 min PCD and ASIGA at 15 min PCD. In accordance with the results, SEM images showed that the lamellae of the fractured specimens of NextDent modified with NDs were different from the smooth surface of the pure specimens. Similarly, for ASIGA specimens, the surface became more lamellated, particularly at 0.5% ND concentration. Moreover, the FTIR results proved that NP incorporation did not alter the chain structures of the nanocomposites but only changed the band intensity.

The addition of SNPs increased the flexural strength of the prototyped resin and it was higher at a concentration of 0.25% than at 0.5%. Similarly, Gad et al. (7) found that SNPs increased the flexural strength of rapidly prototyped denture base resin with higher values recorded at 0.25% than at 0.5%. The nanosized SNPs have a large surface and provide strong bond with the resin matrix due to silanization, thus improving the prototyped resin’s flexural strength (8, 20). However, at high concentrations, clustering of SNPs, as shown in the SEM images, could cause a reduction of flexural strength. These clusters act as stress concentration areas where cracks are initiated and propagated (20). Moreover, the increased concentration of SNPs might increase the viscosity of the printing resin and prevent proper light penetration during polymerization, thus resulting in decreased strength (7). Based on the SEM findings, a concentration of 0.5% showed fewer irregularities with some clustering appearing, compared with 0.25%. This was proved in previous studies which recommended the addition of a low concentration of SNPs (7, 37). Another explanation for the decreased flexural strength with increasing SNP concentrations is the low density of SNPs when compared with other metal oxides (37).

Several factors can affect the mechanical properties of nanocomposites including the type, shape, size, and concentration of the added nanoparticles in addition to the bond strength with the resin (38). Although both the nanoparticles increased the flexural strength, SNPs resulted in higher strength than NDs at each PCD and concentration. Furthermore, the flexural strength was increased with a higher concentration of NDs, while for SNPs, a lower concentration (0.25%) resulted in higher flexural strength. SNPs have a low density, thus the number of particles per unit area is higher than other metal oxide nanoparticles at the same concentration. Accordingly, the addition of SNPs is recommended at low concentrations to avoid particle agglomerations that occur at high concentrations which adversely affect the flexural strength (37).

The addition of NDs resulted in a reduction in the elastic modulus of both materials (ASIGA and NextDent). The elastic modulus is defined as the flexibility of a material within the elastic range (39). Denture base materials are required to possess adequate elastic modulus to avoid permanent deformation that might occur due to continuous stress caused by mastication (40). A previous study tested the impact of ND addition on the flexural properties of heat-polymerized PMMA at concentrations 0.1%, 0.25%, and 0.5% and reported a reduction of elastic modulus at concentrations above 0.1% but it was not statistically significant (19). Nevertheless, the lowest reported value in this study was above 2,000 GPa, which is the accepted elastic modulus for denture base polymers, as recommended by American Dental Association (ADA) specification No. 12 (41).

The elastic modulus of both tested rapidly prototyped resins was not altered by the incorporation of a low SNP concentration (0.25%). However, at 0.5%, the elastic modulus only increased in NextDent resin at 15 min PCD and was decreased in ASIGA resin. Alzayyat et al. (20) found that the elastic modulus of heat-polymerized PMMA was the highest at the lowest concentration of SNPs, 0.05%, and decreased at 0.25% and 0.5%.

The flexural strength was positively correlated with an increase in PCD. The highest flexural strength was recorded at 90 min PCD for both materials for all the different tested nanoparticles and concentrations. The elastic modulus was also increased with an increase in PCD. Previous studies have reported that increasing the PCD increases the strength of prototyped resins (15, 27, 42). An increased PCD reduces the amount of residual monomer and results in complete polymerization, leading to improved strength (27, 42).

The results in our study showed variation in the flexural properties between the tested materials, indicating the significant effect of material type on the flexural properties of rapidly prototyped denture base resins. ASIGA pure resin showed higher flexural strength than pure NextDent resin. This result is in agreement with a previous finding (11). However, after the addition of nanoparticles (NDs and SNPs), the flexural strength and elastic modulus of the NextDent resin were higher than the ASIGA resin at each concentration and PCD. Recently Al Gahmdi et al. (31) reported comparable results with pure ASIGA resin having a higher flexural strength than NextDent resin while after the addition of titanium dioxide nanoparticles, NextDent resin had higher strength than ASIGA resin. The differences between the ingredients of the tested materials and the printers used could be the reason for this variation.

This study tested the effect of NPs and PCD on the flexural strength and elastic modulus of RP denture base resins. Increasing the PCD positively increased the strength of RP resin, as reported in the literature, but the effect of PCD with the addition of NPs has not been tested before. A recent review stated that there is an enhancement of the mechanical and antimicrobial characteristics of 3D-printed resin with the addition of NPs (43). However, studies testing the addition of NPs to 3D-printed denture resin in comparison with heat-polymerized PMMA are still scarce. The effect of SNPs on prototyped denture base resin was tested previously in one study, following the post-curing time recommended by the manufacturer (7). While the influence of NDs on prototyped dentures has not been tested before, it has been tested in 3D-printed orthodontic appliances (21). Therefore, this study aimed to investigate the combined effect of PCD on RP denture base resin with the addition of NDs or SNPs.

The prolonged PCD and addition of nanoparticles increased the flexural strength of both tested resins. Moreover, the effect of the two variables together significantly increased the flexural strength of the tested materials. These results could be beneficial in improving the mechanical strength of RP denture base resins, thus increasing their long-term clinical use.

The present study tested different concentrations of two types of nanoparticles on the flexural strength and elastic modulus of prototyped denture base materials. The specimens were artificially aged by thermal cycling prior to testing to imitate the thermal stress applied on the denture base intraorally. Some limitations of the study include using bar-shaped specimens that do not mimic denture configurations and the absence of other intraoral factors including saliva, oral flora, various pH values, and masticatory forces. Therefore, further *in vivo* studies are required to verify the present results. Finally, the biocompatibility of rapidly prototyped resin with added nanoparticles needs to be tested.

## 5 Conclusions

The flexural properties of rapidly prototyped denture base resins were increased with the addition of NDs and SNPs and by increasing the post-curing duration. SNPs increased the flexural strength at lower concentrations (0.25%), while for NDs, the flexural strength was increased with higher concentrations. Using NDs and low concentrations of SNPs with an extended post-curing duration is recommended for rapidly prototyped denture base fabrication.

## Data Availability

The original contributions presented in the study are included in the article/Supplementary Material, further inquiries can be directed to the corresponding author.
